# Enhancement of the critical current density in FeO-coated MgB_2_ thin films at high magnetic fields

**DOI:** 10.3762/bjnano.2.89

**Published:** 2011-12-14

**Authors:** Andrei E Surdu, Hassan H Hamdeh, I A Al-Omari, David J Sellmyer, Alexei V Socrovisciuc, Andrei A Prepelita, Ezgi T Koparan, Ekrem Yanmaz, Valery V Ryazanov, Horst Hahn, Anatolie S Sidorenko

**Affiliations:** 1 Institute of Electronic Engineering and Nanotechnologies “D.Ghitu” ASM, MD2028, Chisinau, Moldova; 2 Department of Physics, Wichita State University, Wichita, Kansas 67260, USA; 3 Karadeniz Technical University, Physics Department, 61080, Trabzon, Turkey; 4 Institute for Solid State Physics, Russian Academy of Sciences, 132432 Chernogolovka, Russia; 5 Institute of Nanotechnology, Karlsruhe Institute of Technology, D-76021 Karlsruhe, Germany

**Keywords:** critical current, magnesium diboride, nanoparticles, pinning, superconductivity

## Abstract

The effect of depositing FeO nanoparticles with a diameter of 10 nm onto the surface of MgB_2_ thin films on the critical current density was studied in comparison with the case of uncoated MgB_2_ thin films. We calculated the superconducting critical current densities (*J*_c_) from the magnetization hysteresis (*M*–*H*) curves for both sets of samples and found that the *J*_c_ value of FeO-coated films is higher at all fields and temperatures than the *J*_c_ value for uncoated films, and that it decreases to ~10^5^ A/cm^2^ at *B* = 1 T and *T* = 20 K and remains approximately constant at higher fields up to 7 T.

## Introduction

After the discovery of superconductivity in MgB_2_ [1], this material became attractive for researchers all over the world not only because of its special physical properties but also due to its possible technical applications. This material, with a hexagonal crystal structure and a critical temperature of *T*_c_ = 39 K, raised a lot of questions about its transport properties. This strong type-II superconductor has a fairly high critical current density in zero magnetic field, i.e., up to *J*_c_ ~ 1.6 × 10^7^ A/cm^2^ at 15 K [2]. This superconducting parameter makes it a very attractive candidate to replace Nb in various superconducting devices, namely for devices operating at temperatures around 20 K, which are attainable in low-cost cryocoolers. However, the dramatic fall of the critical current in an external magnetic field at temperatures around 20 K limits the possible use of magnesium diboride in engineering applications. Therefore, for a wide-scale technical application of MgB_2_ it is necessary to solve the problem of the enhancement of its critical current in an external magnetic field.

## Results and Discussion

There have been many attempts to solve the above-mentioned problem relating to the decay of the critical current in an external magnetic field. Various research teams have tried to increase the critical current density either by doping MgB_2_ with various substances (carbon [3], aluminium [4], etc.) or by adding nanoparticles of SiC [5], nanodiamonds [6], etc. As we can conclude from these works, the highest value of the critical current in the zero magnetic field is *J*_c_ ~ 10^6^ A/cm^2^ in a temperature range of 5–25 K, and the highest value at a magnetic field of 8 T is *J*_c_ ~ 10^4^ A/cm^2^ at 4.2 K; no significant increase was reported at higher temperatures and higher fields (*J*_c_ ~ 10^2^ A/cm^2^ at 6 T and 20 K).

In our previous study of the resistive transitions of MgB_2_ films in an external magnetic field [7], we showed that the rapid decrease of the activation energy of the flux flow for MgB_2_ in the field region *B* > 1 T represents a dramatic loss of the current-carrying abilities of this superconductor due to the weakening of the flux-line pinning with increasing magnetic field. The problem to be solved is how to increase the pinning force and to overcome the dramatic dropdown of the critical current in a strong external magnetic field for this superconducting material. For this purpose, a novel method of depositing self-assembled nanoparticles with various distance parameters onto the sample surface was used in our experiments. Our suggestion for tackling this problem was the following: The presence of magnetic nanoscale pinning centers should increase the pinning force due to the magnetic interaction between nanoparticles and vortices, which was calculated in [8]. Based on these calculations one can choose the appropriate diameter of nanoparticles to efficiently increase the magnetic pinning force. One more argument is that ferromagnets strongly suppress superconductivity, and even a small ferromagnetic region can be a strong pin, as was confirmed in experiments with NbTi wires containing nanometer-sized arrays of Ni pins [9]. We placed the ferromagnetic nanoparticles on the surface, instead of in the volume of the films, in order to avoid the strong suppression of the critical temperature of the film. The proximity effect of nanoparticles has a very small effect on the superconducting properties of our MgB_2_ thin films, reducing their critical temperature by about 1 K. Taking into account these ideas, we studied the effect of nanoparticles deposited onto the surface of MgB_2_ thin films on the transport properties of these films.

In order to carry out the proposed research, MgB_2_ films with a thickness of about 600 nm were prepared on the MgO (100) substrates by using a “two-step” synthesis technology similar to the method described in detail in [10]. The X-ray diffraction patterns show the high quality of the prepared polycrystalline MgB_2_ films, with the parameters of the MgB_2_ unit cell being *a* = 3.08 Å, *c* = 3.53 Å, which are close to the values for *a* and *c* of bulk MgB_2_, as was investigated in detail in our previous work [11].

Thin MgB_2_ film samples with dimensions ~4.5 × 5.0 mm^2^ were used in our work. These samples were obtained by cutting a MgB_2_ film into two similar pieces: One 4.38 × 5.18 mm^2^ piece was covered with FeO nanoparticles, which had a diameter of 10 nm, by spin coating at 4000 rpm centrifuge, and the other 4.28 × 4.84 mm^2^ piece remained uncoated for comparison measurements. The Ginzburg–Landau coherence length of our films was obtained from the slope of the upper critical magnetic field measurements, *B*_c2_(*T*), close to the critical temperature, resulting in *ξ*_GL_(0) = 3.0 nm [12].

We measured the magnetization hysteresis (*M*–*H*) curves of the FeO-covered and uncovered MgB_2_ films at *H* perpendicular to the sample surface at various temperatures from 4.2 K to 20 K. All the magnetization measurements were performed in a superconducting quantum interference device (SQUID) magnetometer (Quantum Design, Magnetic Property Measurement System, MPMS-XL). The SQUID magnetometer has a sensitivity of l0^−8^ emu and operates in the temperature range 1.9–400 K, with magnetic fields up to 7 T; it has a high field uniformity of 0.01% over 4 cm. Pd was used as a standard for the SQUID magnetometer.

**Figure 1:**
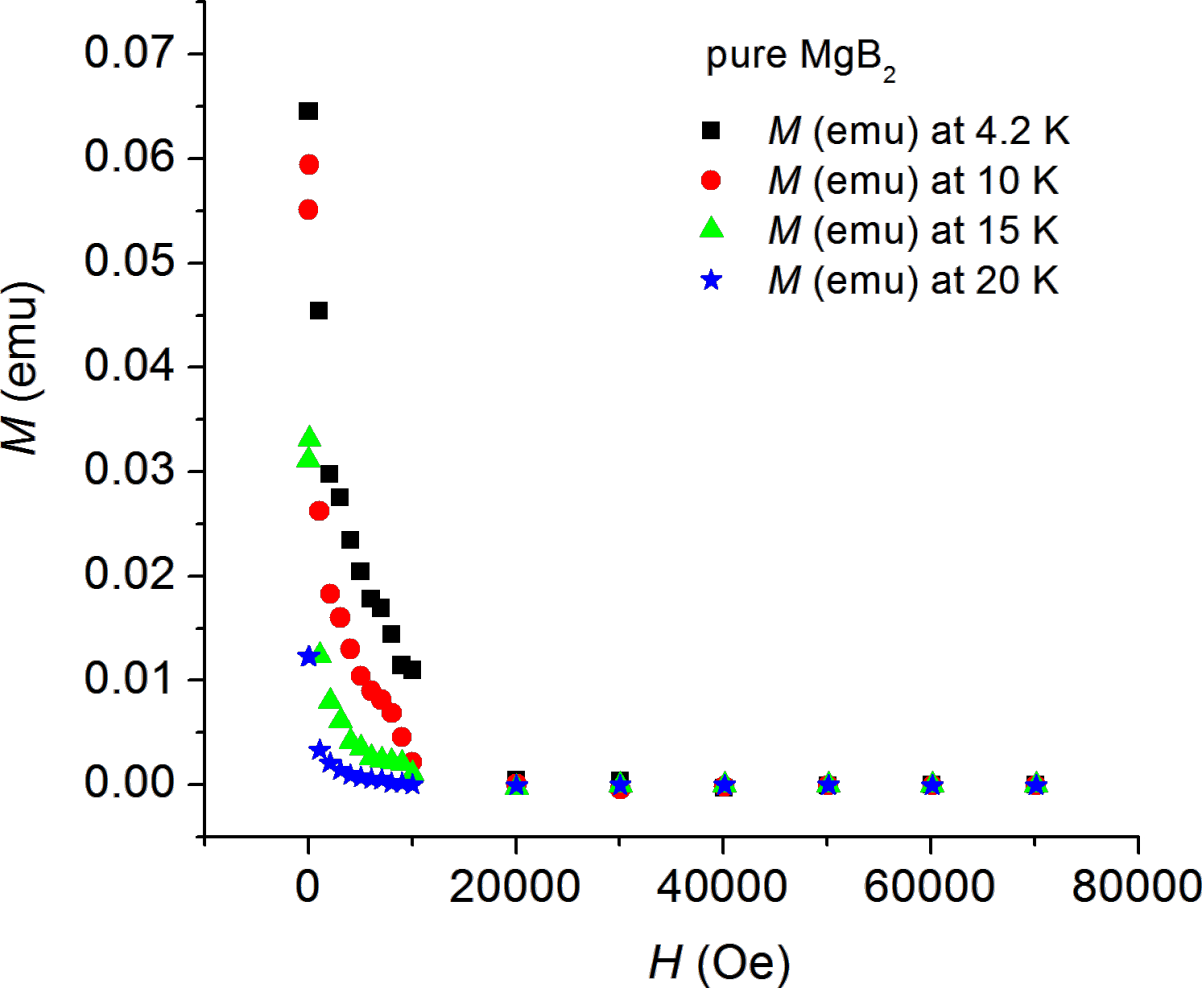
Magnetization hysteresis (*M–H*) curves for the pure MgB_2_ sample at various temperatures: 4.2 K, 10 K, 15 K, and 20 K.

**Figure 2:**
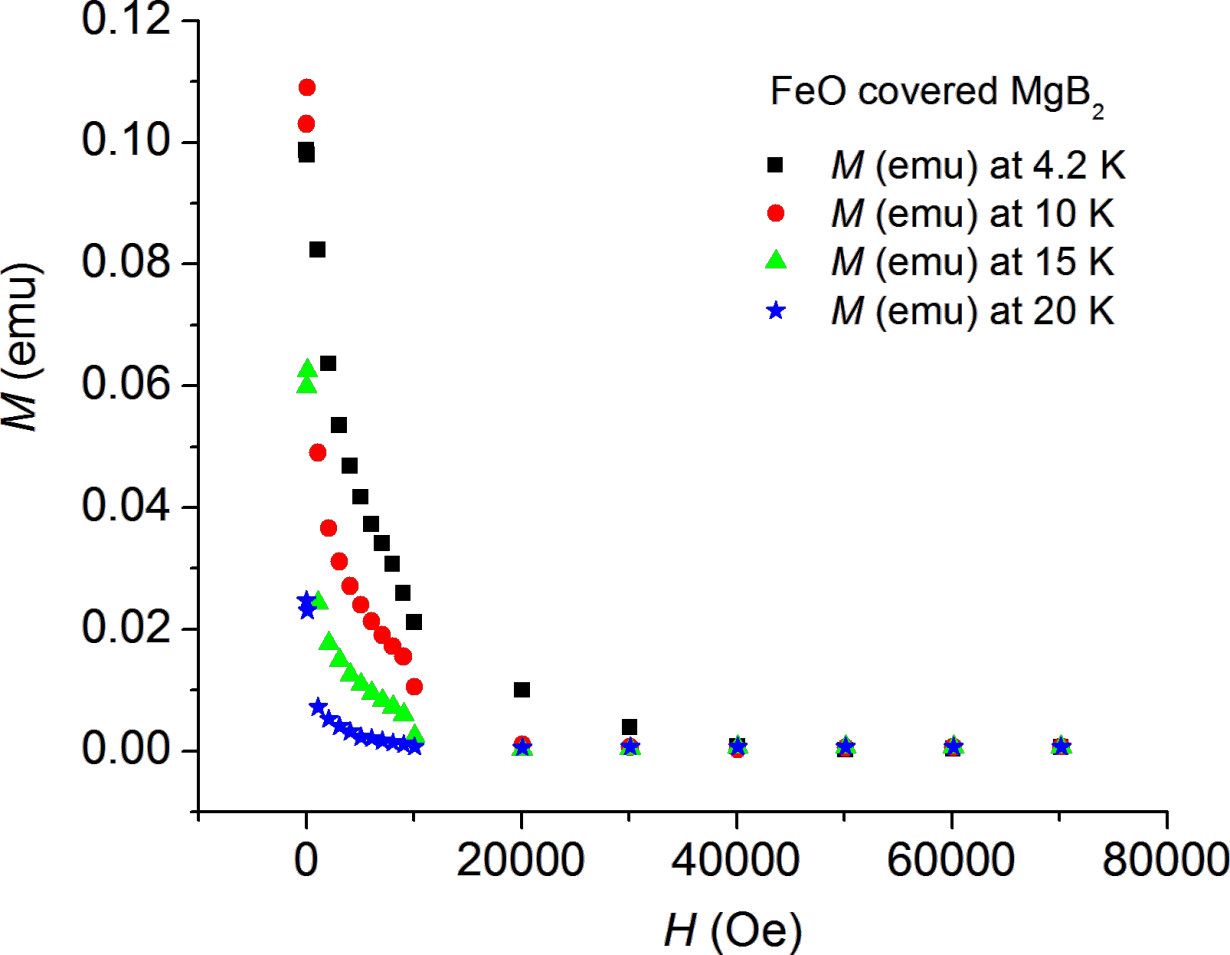
Magnetization hysteresis (*M–H*) curves for the FeO-covered MgB_2_ sample at various temperatures: 4.2 K, 10 K, 15 K, and 20 K.

The magnetic measurements were first done for the substrate by itself and the magnetic moments of the substrate were subtracted (after mass normalization) from the magnetic signal of each of the MgB_2_ thin-film samples. The measured (*M–H*) curves are shown in Figure 1 and Figure 2.

At first glance, the values of the magnetic moment of the sample covered with FeO nanoparticles are considerably higher than the respective values of the uncovered sample; as is especially clear to see in the field range 0–4 T at 4.2 K.

**Figure 3:**
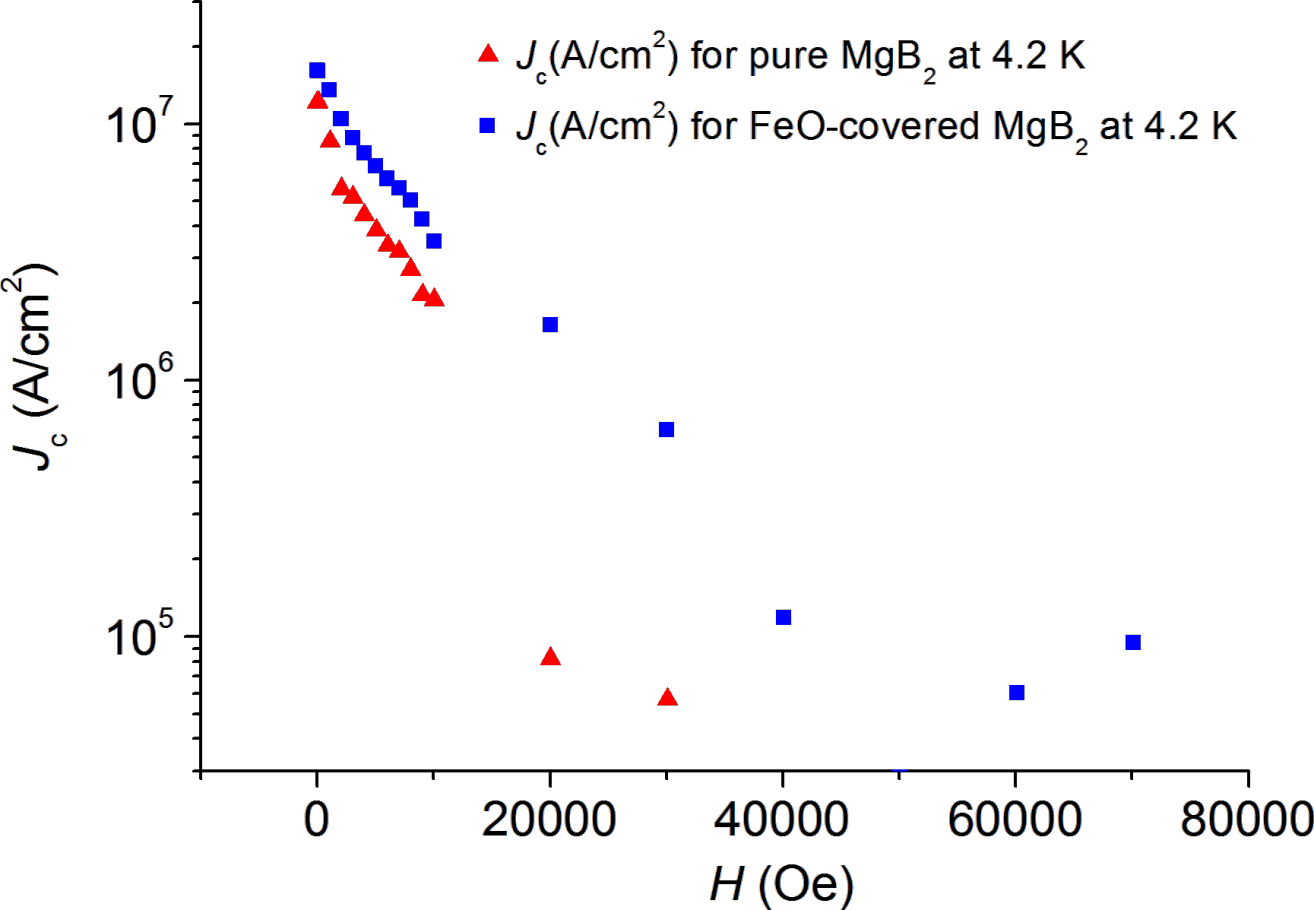
Magnetic-field dependence of the critical current density *J*_c_ for the pure (red triangles) and for the FeO-covered (blue squares) MgB_2_ samples at 4.2 K.

The curves in Figure 3 to Figure 6 show the values of the critical current density *J*_c_ as a function of the applied magnetic field at various temperatures, which are estimated from the *M*–*H* curves by using the Bean’s critical-state model formula: *J*_c_ = 30 Δ*M/r*, where Δ*M* is the height of the *M*–*H* curve. We choose the effective sample size *r* as the radius of the circle whose total area is the same as the sample size, i.e., by using π*r*_1_^2^ = 4.28 × 4.84 mm^2^ for the pure sample and π*r*_2_^2^ = 4.38 × 5.18 mm^2^ for the FeO-covered sample. Thus, we used the effective sample sizes *r*_1_ = 2.57 mm and *r*_2_ = 2.69 mm, which are orders of magnitude larger than the grain size.

**Figure 4:**
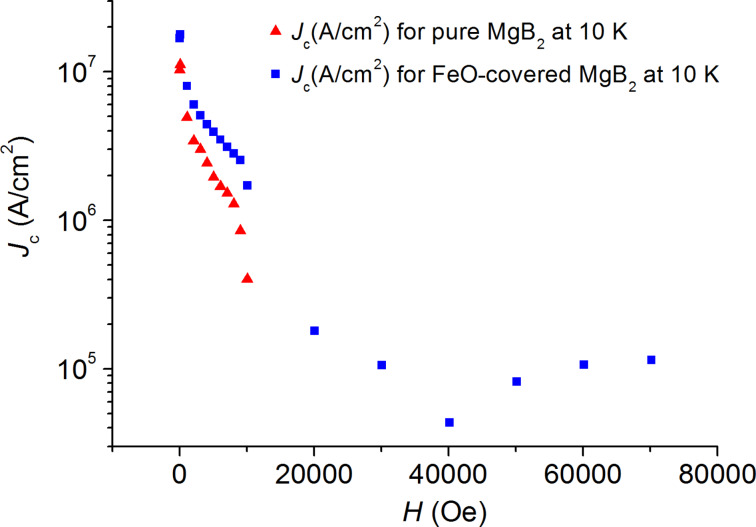
Magnetic-field dependence of the critical current density *J*_c_ for the pure (red triangles) and for the FeO-covered (blue squares) MgB_2_ samples at 10 K.

**Figure 5:**
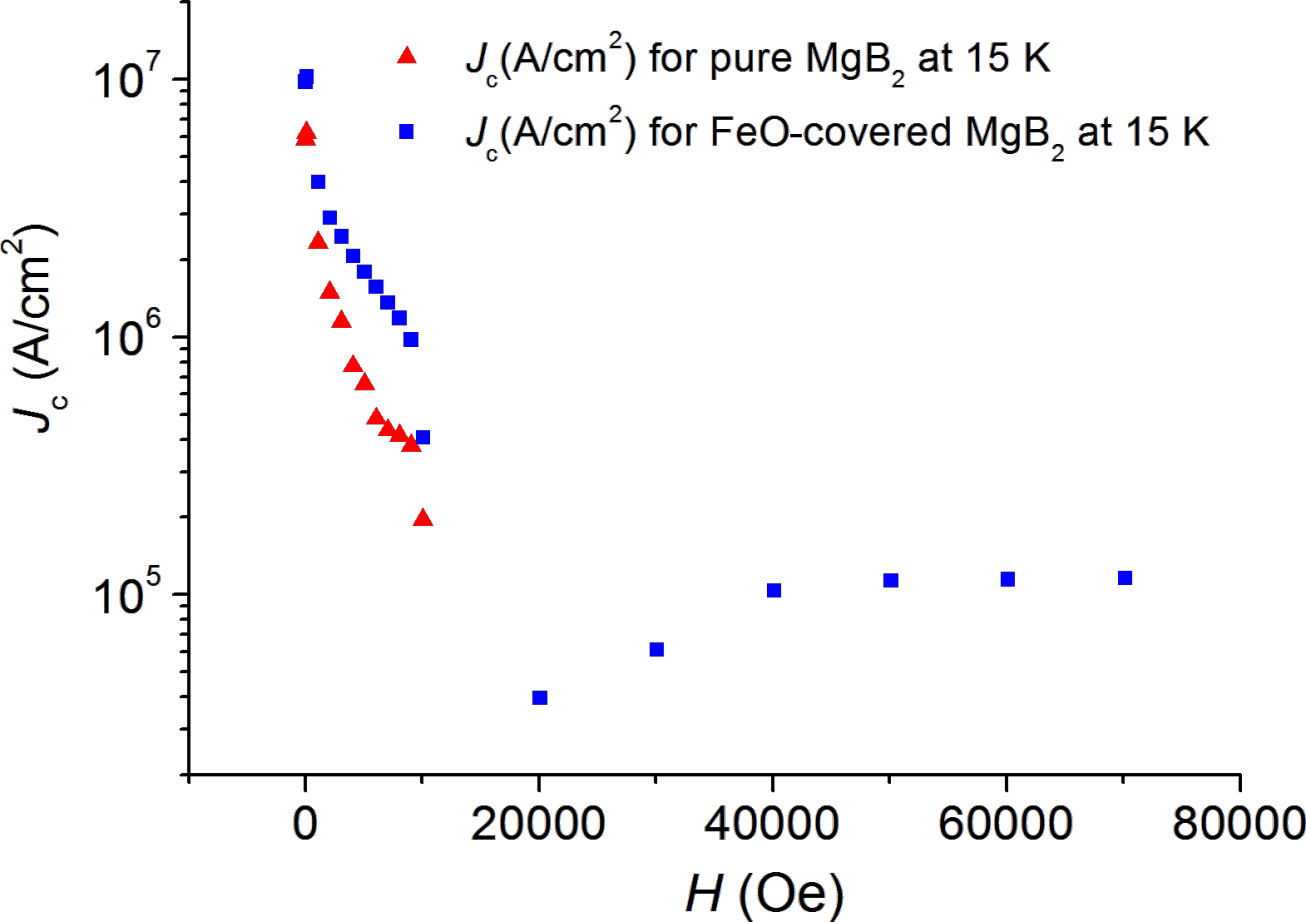
Magnetic-field dependence of the critical current density *J*_c_ for the pure (red triangles) and for the FeO-covered (blue squares) MgB_2_ samples at 15 K.

**Figure 6:**
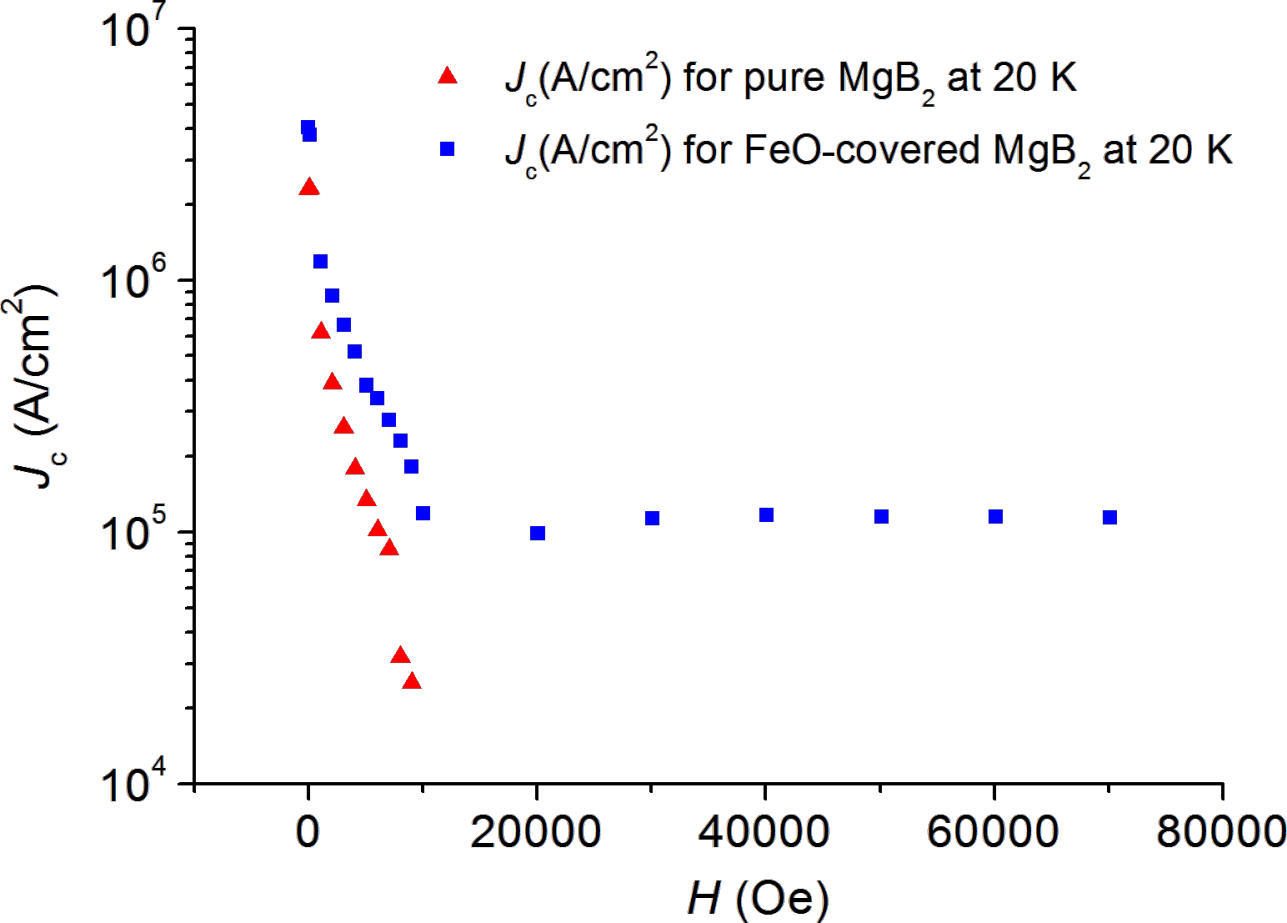
Magnetic-field dependence of the critical current density *J*_c_ for the pure (red triangles) and for the FeO-covered (blue squares) MgB_2_ samples at 20 K.

Figure 3 shows that after FeO coating of the sample the values of *J_c_* at *T* = 4.2 K increased by one order of magnitude in the field range of 1–4 T; the critical current density for the coated sample decreased gradually with an increase of the applied magnetic field and was equal to 10^5^ A/cm^2^ at *B* = 7 T, whereas for the uncoated sample *J_c_* dropped down abruptly and became negligibly small at fields *B* > 3 T.

In the temperature range 10–20 K (Figure 4, Figure 5 and Figure 6) we observe the same behavior of *J_c_* for both samples: *J_c_* was approximately equal to 10^5^ A/cm^2^ for the coated sample in the field range of 3–7 T, whereas the *J_c_* value of the pure sample dropped down and was negligibly small at fields *B* > 1 T.

We believe that the observed effect of an increase of *J_c_* after the coating of the sample with FeO nanoparticles is related to an increase of the pinning force due to the magnetic interaction between nanoparticles and vortices. The obtained value of *J_c_* ~ 10^5^ A/cm^2^ at *T* = 20 K and *B* = 7 T (see Figure 6) is higher than the *J_c_* value of 10^4^ A/cm^2^ at *T* = 5 K and *B* = 7 T, and the *J_c_* value of 10^2^ A/cm^2^ at *T* = 10 K and *B* = 7 T reported recently in [13], which was obtained by doping of MgB_2_ with C_60_.

## Conclusion

In summary, we obtained the *J_c_* dependence on the applied magnetic field from the *M–H* curves for pure MgB_2_ thin films and from films covered with FeO (10 nm nanoparticles). We report a significant increase of the critical current density for the FeO-coated MgB_2_ thin films obtained by the two-step method. After deposition of the nanoparticles the critical temperature of the films decreased by 0.7 K, whereas the *J_c_* value rose to ~10^5^ A/cm^2^ at *T* = 20 K and *B* = 7 T. This value of the critical current density is higher than any previously published in the literature, to our knowledge. As the main result of the present work, we have elaborated a simple method for the enhancement of the flux pinning and the supercurrent-carrying ability of magnesium diboride thin films.
